# Modeling the Interaction between *β*-Amyloid Aggregates and Choline Acetyltransferase Activity and Its Relation with Cholinergic Dysfunction through Two-Enzyme/Two-Compartment Model

**DOI:** 10.1155/2015/923762

**Published:** 2015-08-30

**Authors:** Hedia Fgaier, Ibrahim H. I. Mustafa, Asmaa A. R. Awad, Ali Elkamel

**Affiliations:** ^1^Department of Mathematics and Statistics, University of Guelph, Guelph, ON, Canada N1G 2W1; ^2^Biomedical Engineering Department, Faculty of Engineering at Helwan, Helwan University, Helwan, Cairo 11792, Egypt; ^3^Chemical Engineering Department, University of Waterloo, Waterloo, ON, Canada N2L 3G1

## Abstract

The effect of *β*-amyloid aggregates on activity of choline acetyltransferase (ChAT) which is responsible for synthesizing acetylcholine (ACh) in human brain is investigated through the two-enzyme/two-compartment (2E2C) model where the presynaptic neuron is considered as compartment 1 while both the synaptic cleft and the postsynaptic neuron are considered as compartment 2 through suggesting three different kinetic mechanisms for the inhibition effect. It is found that the incorporation of ChAT inhibition by *β*-amyloid aggregates into the 2E2C model is able to yield dynamic solutions for concentrations of generated *β*-amyloid, ACh, choline, acetate, and pH in addition to the rates of ACh synthesis and ACh hydrolysis in compartments 1 and 2. It is observed that ChAT activity needs a high concentration of *β*-amyloid aggregates production rate. It is found that ChAT activity is reduced significantly when neurons are exposed to high levels of *β*-amyloid aggregates leading to reduction in levels of ACh which is one of the most significant physiological symptoms of AD. Furthermore, the system of ACh neurocycle is dominated by the oscillatory behavior when ChAT enzyme is completely inhibited by *β*-amyloid. It is observed that the direct inactivation of ChAT by *β*-amyloid aggregates may be a probable mechanism contributing to the development of AD.

## 1. Introduction

**Figure 1:**
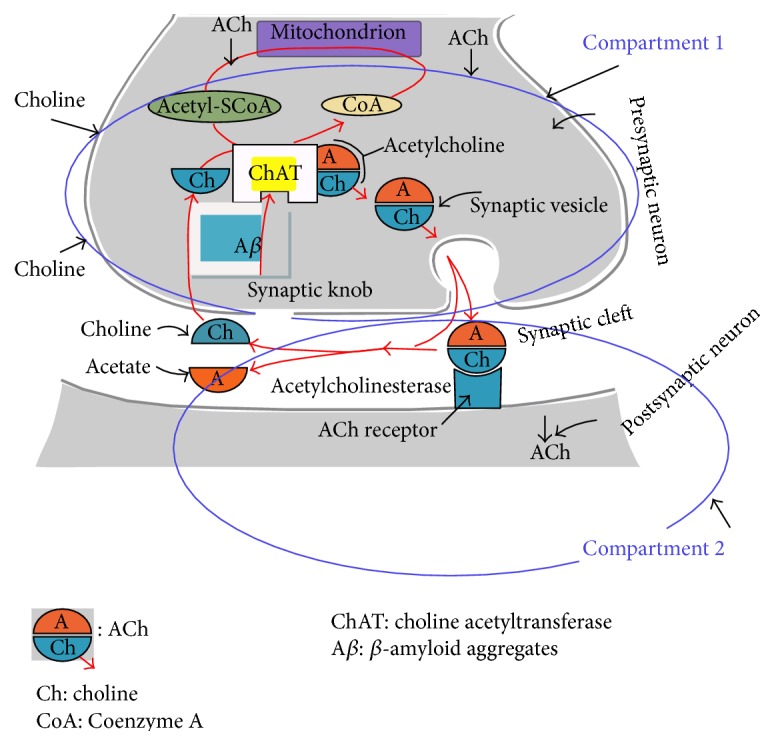
Schematic representation of ChAT catalytic role in ACh synthesis: two-enzyme/two-compartment (2E2C) model [5, 6].

Choline acetyltransferase (ChAT) is a single-strand globular protein and catalyzes biosynthesis reaction of acetylcholine (ACh) neurotransmitter [1]. It was discovered in rabbit brain by Nachmansohn and Machado in 1943 [2]. ChAT is present in two forms in nerve terminal: (i) nonionic membrane-bound and (ii) soluble form. ChAT synthesis occurs in the rough endoplasmic reticulum in cholinergic neuron cells. Then, it is transferred to axon terminal for ACh synthesis via rapid and slow axoplasmic flow [3]. ChAT plays an essential role in cholinergic neuron cells and nonneuron cells. In neuron cells ChAT expression is a special marker for cholinergic system improvement during brain growth and development where ChAT is involved in activation of multiple neuropsychic functions such as memory, cognition, sleep, learning, and movement [4]. Therefore, any disturbance in ChAT levels inside neuron cells could generate serious negative consequences. For example, (i) downregulation of ChAT activity causes serious cholinergic disease such as dementia, schizophrenia, and Huntington's disease [5–7], (ii) ChAT abnormalities in the brain could lead to sudden death in infants [3], and (iii) ChAT inhibition leads to generation of Alzheimer's disease because ChAT is the enzyme that is responsible for ACh synthesis. Therefore, ChAT inactivation results in a decline of ACh levels in brain. Decreasing ACh concentration is the most common early stage features of AD. However, in nonneuron cells ACh generated by ChAT catalytic effect is secreted in extracellular space. ACh in extracellular space is necessary for maintaining homeostasis of cells and for modulating many of the essential cell functions such as cell division, mitosis, cytoskeleton organization, interactions between cells, and maintaining the immune functions [4]. As shown in Figure 1 ChAT is concentrated in the presynaptic neuron of the cholinergic neuron because of its necessity for ACh synthesis [6, 8] where ChAT catalyzes the reaction between choline (taken from extra cellular fluid by high-affinity transporter carrier) and acetyl-CoA (produced during glucose metabolism in mitochondria) to produce ACh; ChAT facilitates the transfer of acetyl moiety from acetyl-CoA to choline to give ACh and enzyme-CoA as indicated in

(1) $$\begin{array}{c} \underset{\text{Choline}}{{\left(CH_{3}\right)}_{3}\overset{+}{N}CH_{2}CH_{2}OH}+\underset{\text{Acetyl coenzyme A}}{CH_{3}COSCoA}\overset{ChAT}{↔}\underset{\text{ACh}}{{\left(CH_{3}\right)}_{3}\overset{+}{N}CH_{2}CH_{2}OCOCH_{3}}+CoASH \end{array}$$

There are various factors that could negatively affect ChAT activity such as excitatory amino acids, reactive oxygen species, and *β*-amyloid aggregates. The most serious defective factor is *β*-amyloid aggregates [9]. Incubation of nanomolar concentration of *β*-amyloid aggregates with 50% cultured cholinergic neurons caused complete ChAT inhibition [10]. ChAT inhibition by *β*-amyloid aggregates induces several cholinergic problems such as memory dysfunction, neuron impairment, and cognition declining. Since ChAT is an important indicator for health of cholinergic neurons and plays a necessary role for activating multiple neuropsychic functions, many researches focused on ChAT investigation such as Mustafa et al. [5, 6], Nunes-Tavares et al. [11], and Waser et al. [12].

The main goal of this work is to investigate the effect of wide range of concentrations of *β*-amyloid aggregates as inhibitor on ChAT activity in brain tissues and the consequences on the cholinergic neurocycle behavior such as concentrations of ACh, choline levels, acetate levels, and the rate of ACh synthesis and ACh hydrolysis in compartment 1 and compartment 2, respectively. In this study three different kinetic mechanisms describing the interaction between *β*-amyloid aggregates and ChAT activity are proposed and the results of each mechanism are compared to each other in order to understand how *β*-amyloid aggregates could affect ChAT activity. The rate of ACh synthesis catalyzed by ChAT and affected by *β*-amyloid inhibitor and derived from each kinetic mechanism is incorporated into the two-enzyme/two-compartment model presented by Mustafa et al. [5, 6] to account for the effect of *β*-amyloid aggregates.

## 2. Formulation of Interaction between ***β***-Amyloid Aggregates and ChAT Kinetics through 2E2C Model

**Figure 2:**
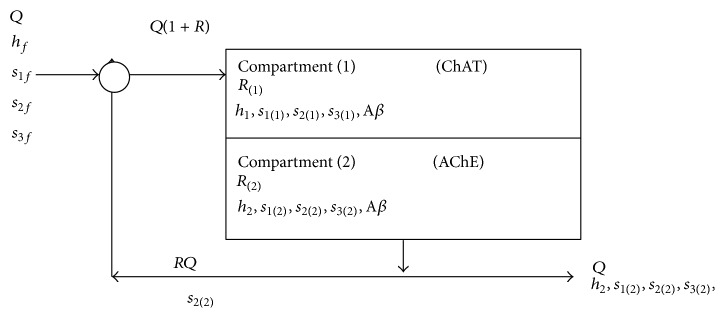
Schematic representation for two-enzyme/two-compartment model.

The ACh neurocycle is shown in Figure 1 where the presynaptic neuron represents the plant for ACh synthesis and contains the enzyme ChAT, choline, acetyl-CoA, ACh, and *β*-amyloid aggregates (A*β*). The presynaptic neuron is considered compartment 1 while both synaptic cleft and postsynaptic neuron represent compartment 2. It is observed that choline is the only component produced from the hydrolysis which is recycled to compartment 1 and reused for ACh synthesis [5]. Tucek [13] showed that both ACh and acetyl-CoA are synthesized in compartment 1.  Figure 2 shows a simple form of the feedback model of ACh cholinergic neurocycle shown in Figure 1. Every compartment is considered a constant volume and isothermal continuous stirred tank reactor (CSTR) with a constant flow rate and constant recycle ratio. Also, the two compartments are separated by a permeable membrane. *β*-amyloid aggregates interact with ChAT inside compartment 1. Nunes-Tavares et al. [10] reported that there is a strong relation between *β*-amyloid peptides and cholinergic dysfunction which is one of the main symptoms of Alzheimer's disease (AD). Furthermore, *β*-amyloid could inhibit the activity of the enzyme ChAT leading to reduction in the levels of ACh and memory impairment [10]. ChAT activity could be completely reduced significantly with *β*-amyloid.

### 2.1. Model Assumptions

All assumptions made for investigating the effect of *β*-amyloid aggregates compatible with literature such as those cited by Mustafa et al. [5, 6] and Mustafa et al. [14, 15] are considered in this work. More justifications and assumptions are clarified below:

(1) The presynaptic of cholinergic nerve terminal is described by compartment 1. Inside this compartment ACh is synthesized by the reaction of choline and acetyl-CoA in the presence of catalytic effect of ChAT enzyme.
(2) Postsynaptic cleft of neurons together with synaptic cleft are considered as compartment two. Both postsynaptic and synaptic clefts are unified to be in one compartment instead of two or three because both of them are harmonized and are interactive and also to simplify the calculations in solving the model particularly when the dimensionality is too high.
(3) Both compartments are assumed to be divided with a permeable membrane.
(4) The internal masses transfers in compartment one between synaptic vesicles and cytoplasm and in compartment two between ACh and postsynaptic receptors are neglected because every compartment is assumed to be homogenous.
(5) All matters in the presynaptic neuron are transported to the postsynaptic cleft via passive diffusion where all concentrations in compartment 1 should be higher than that in compartment 2; however, choline uptake from the postsynaptic cleft to the presynaptic neurons is performed by facilitated diffusion via high-affinity choline uptake transporters.
(6) It is observed that concentrations of all state variables in compartment two are the average of concentrations in postsynaptic and synaptic cleft.
(7) Temperature is considered constant where the system is assumed to be isothermal and there is no effect for any temperate changes on the system.
(8) Transport of *β*-amyloid aggregates from compartment 1 to compartment 2 is neglected. To justify, it is noted that transport of *β*-amyloid aggregates from compartment 1 to compartment 2 is by passive diffusion and the transport rate is extremely lower than that from the cytoplasm to compartment 1. The latter transport (from cytoplasm to compartment 1) addresses the blood brain barriers according to saturable mechanism such as Monod and Michaelis-Menten kinetics [16–18].
(9) The initial concentration of *β*-amyloid aggregates is assumed to be zero.
(10) Feed stream rate to compartment one and outlet flow from compartment two are considered to be constant with a value of *q* (m^3^/sec).

### 2.2. Competitive Inhibition of ChAT in Two-Enzyme/Two-Compartment Model

ChAT and AChE are the two cholinergic enzymes considered in the 2E2C model. ChAT is responsible for synthesis of ACh in compartment 1 of the 2E2C model, while AChE catalyzes the degradation of ACh in compartment 2. It is found that incorporation of *β*-amyloid aggregates into rat brains* in vivo* [19] and at nano- to picomolar concentrations into SN56 cell line* in vitro* [20] shows significantly a decrease in ChAT activity and ACh production. However, detection of change in AChE activity was readily observed and reported [21]. This finding suggests that *β*-amyloid aggregates may act as a general modulator of ChAT enzyme activity leading to a reduction in ACh synthesis [22].

The exact mechanisms of ChAT modulation by *β*-amyloid aggregates are still currently unknown. It is possible that *β*-amyloid aggregates may directly inhibit ChAT activity by competitively binding to active sites or perhaps limit the activity of secondary proteins responsible for ChAT synthesis and regulation [23]. Since level of intracellular ChAT is generally maintained at a concentration much higher than that of the reactants for ACh production [24], modification of the 2E2C model to incorporate inhibition of ChAT activity is required to understand the mechanisms of the interaction between ChAT and *β*-amyloid aggregates.

**Figure 3:**
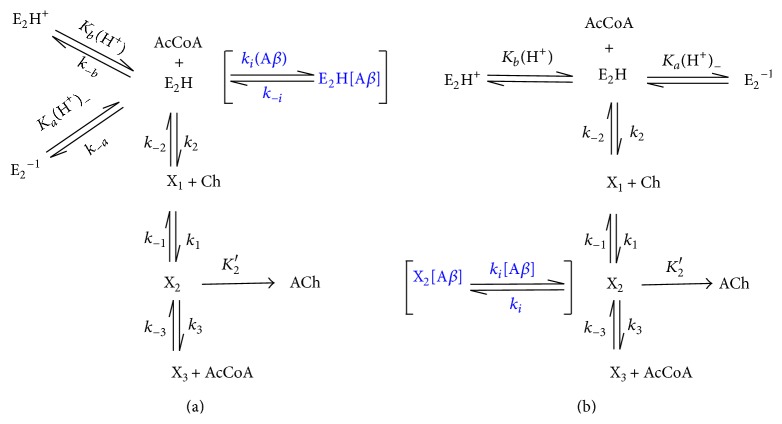
Possible competitive inhibition mechanisms for *β*-amyloid ([A*β*]) during pH-dependent ChAT synthesis of ACh (areas of inhibition are displayed in square brackets). The reaction route occurs in the vertical direction. E_2_H is the active form of the enzyme; E_2_H^+^ and E_2_^−1^ are the protonated and deprotonated inactive forms. X_1_, X_2_, and X_3_ are enzyme intermediate complexes. (a) *β*-amyloid competitively binds to active enzyme complex E_2_H; (b) *β*-amyloid competitively binds to enzyme intermediate X_2_.

To examine the phenomenon of ChAT activity inhibition by *β*-amyloid aggregates in a simpler model, *β*-amyloid aggregate is considered to act directly as a competitive inhibitor of ChAT enzyme.  Figure 3 illustrates a modified version of pH-dependent enzyme synthesis reaction model used in the two-enzyme/two-compartment model. The modification (represented in square brackets) takes into account two possible pathways in which *β*-amyloid may bind competitively to intermediate complexes, thereby inhibiting their ability to progress to the main reaction direction (in the vertical pathway) towards ACh synthesis. In such case *β*-amyloid aggregates (A*β*) may bind directly to the active form of the enzyme E_2_H to generate the inactive E_2_H [A*β*] complex (Figure 3(a)), thus limiting the availability of active enzymes to carry out ACh synthesis and decrease net enzyme activity. Similarly, it is also possible that *β*-amyloid may bind to the enzyme intermediate complex X_2_, thereby preventing ACh release from the complex (Figure 3(b)).

*β*-amyloid aggregates combine with ACh molecules in the presynaptic neuron (compartment 1) to form complexes which are unable to play the same role of ACh [23]. Therefore, the rate of decline in ACh is sensitive to the rate of generation of *β*-amyloid aggregates. To describe the relevant complex processes mathematically, the following ordinary differential equation is proposed to account for the reduction of ACh production in compartment 1 (*s*_1(1)_) due to the interaction with *β*-amyloid aggregates:

(2) $$\begin{array}{c} \frac{ds_{1\left(1\right)}}{dT}=-K_{bA1}s_{1\left(1\right)}A\beta . \end{array}$$

It is assumed that the rate of ACh reduction is proportional to the concentration of *β*-amyloid (A*β*) aggregates and ACh in compartment 1 where *K*_*bA*1_ is constant. In addition, the sophisticated mathematical model is developed to investigate the interaction between *β*-amyloid aggregates and ChAT. The model takes into consideration generation and clearance of A*β* aggregates [25, 26]. It is assumed that the level A*β* is kept via the balancing of a zero-order generation rate (*K*_*L*2_) and first-order clearance processes (*K*_*L*3_ *s*_1(1)_ + *K*_*L*4_A*β*). The accumulation of A*β* is described by

(3) $$\begin{array}{c} \frac{dA\beta }{dT}=K_{L2}-K_{L3}s_{1\left(1\right)}-K_{L4}A\beta , \end{array}$$

where the term (*K*_*L*2_) describes the generation of A*β* aggregates from amyloid precursor protein, the term (−*K*_*L*3_ *s*_1(1)_) describes the impact of ACh levels in the presynaptic neuron on the rate of formation of A*β* aggregates [27, 28] and the term (−*K*_*L*4_A*β*) represents the reduction in A*β* aggregates levels due to the enzymatic hydrolysis and diffusion in the neuronal membranes [28].

The numerical derivation of the ChAT rate equation for the modified pH-dependent ACh synthesis with competitive inhibition of *β*-amyloid is shown in Sections 3, 4, and 5. Equations (1) and (2) in addition the new ChAT rate equations (*r*_1_) derived from each kinetic mechanism are incorporated into the model built by Mustafa et al. [5, 6] to give our model as shown in Table 1.

## 3. Kinetic Mechanism 1: Competitive Inhibition of A***β*** with All Species Activated Enzyme Complex E_2_H

### 3.1. Formulation of Kinetic Mechanism 1

The synthesis rate for competitive inhibition of A*β* aggregates with activated enzyme complex E_2_H can be derived as follows.

X_1_ and X_2_ are related to each other through the following equation:

(4) $$\begin{array}{c} X_{1}\left(Ch\right)k_{1}=X_{2}k_{-1}\\ \text{or}\;\;X_{1}=\frac{K_{1}}{Ch}X_{2}, \end{array}$$

where *K*_1_ = *k*_−1_/*k*_1_.

By the assumption of rapid equilibrium, E_2_H and X_1_ can be related by

(5) $$\begin{array}{c} k_{2}\left(AcCoA\right)E_{2}H=k_{-2}X_{1}\\ or\;E_{2}H=\frac{K_{1}K_{2}}{\left(AcCoA\right)\left(Ch\right)}X_{2}, \end{array}$$

where  *K*_2_ = *k*_−2_/*k*_2_.

By rapid equilibrium assumption, E_2_H[A*β*] complex can be expressed as

(6) $$\begin{array}{c} k_{i}E_{2}H\left(A\beta \right)=k_{-i}E_{2}H\left[A\beta \right]\\ \text{or}\;\;E_{2}H\left[A\beta \right]=K_{i}\left(A\beta \right)\left(E_{2}H\right), \end{array}$$

where *K*_*i*_ = *k*_*i*_/*k*_−*i*_.

From the ionization of E_2_H,

(7) $$\begin{array}{c} E_{2}H\left(H^{+}\right)k_{-b}=E_{2}H^{+}k_{b}\\ \text{or}\;\;E_{2}H^{+}=\frac{\left(E_{2}H\right)\left(H^{+}\right)}{K_{b}}. \end{array}$$

From the deionization of E_2_H,

(8) $$\begin{array}{c} E_{2}^{-}\left({H_{}}^{+}\right)k_{a}=E_{2}Hk_{-a}\\ or\;\;E_{2}^{-}=\frac{K_{a}\left(E_{2}H\right)}{\left(H^{+}\right)}. \end{array}$$

From (4), (5), (6), (7), and (8) we get

(9) $$\begin{array}{c} E_{t2}=E_{2}H+{E_{2}}^{-}+E_{2}{H_{1}}^{+}+E_{2}H\left[A\beta \right]=\frac{K_{2}K_{1}}{\left(AcCoA\right)\left(Ch\right)}\left(1+\frac{K_{a}}{{H_{}}^{+}}+\frac{{H_{}}^{+}}{K_{b}}+\left(A\beta \right)K_{i}\right)X_{2}. \end{array}$$

X_2_ and X_3_ are related by

(10) $$\begin{array}{c} X_{3}=\frac{X_{2}}{K_{3}\left(AcCoA\right)}, \end{array}$$

where *K*_3_ = *k*_−3_/*k*_3_.

Since E_*t*_ = E_*t*2_ + X_1_ + X_2_ + X_3_,

(11) $$\begin{array}{c} E_{t}=X_{2}\left(\frac{K_{2}K_{1}}{AcCoA\left(Ch\right)}\left(1+\frac{K_{a}}{{H_{1}}^{+}}+\frac{{H_{1}}^{+}}{K_{b}}+\left(bA\right)K_{i}\right)+\frac{K_{1}}{Ch}+1+\frac{1}{K_{3}\left(AcCoA\right)}\right)\\ \text{or}\;\;X_{2}=\frac{E_{t}\left(Ch\right)\left(AcCoA\right)}{\left(K_{2}K_{1}\left(1+\left(K_{a}/{H_{1}}^{+}\right)+\left({H_{1}}^{+}/K_{b}\right)+\left(A\beta \right)K_{i}\right)+K_{1}\left(AcCoA\right)+Ch\left(AcCoA\right)+Ch/K_{3}\right)}\\ X_{2}=\frac{E_{t}\left(Ch\right)\left(AcCoA\right)}{\left(K_{2}K_{1}\left(1+\left(K_{a}/{H_{1}}^{+}\right)+\left({H_{1}}^{+}/K_{b}\right)+\left(A\beta \right)K_{i}\right)+K_{1}\left(AcCoA\right)+Ch\left(AcCoA\right)+Ch/K_{3}\right)}\\ h_{1}=\frac{H_{1}^{+}}{K_{a}}\\ X_{2}=\frac{E_{t}\left(Ch\right)\left(AcCoA\right)/\left(S_{2ref}S_{3ref}\right)}{\left(\left(K_{2}K_{1}/S_{2ref}S_{3ref}\right)\left(1+\left(1/h_{1}\right)+\left(K_{a}/K_{b}\right)h_{1}+\left(A\beta \right)K_{i}\right)+\left(K_{1}/S_{2ref}S_{3ref}\right)\left(AcCoA\right)+\left(Ch\left(AcCoA\right)/S_{2ref}S_{3ref}\right)+\left(Ch/K_{3}\right)\left(1/S_{2ref}S_{3ref}\right)\right)}\\ X_{2}=\frac{E_{t}\left(Ch\right)\left(AcCoA\right)/\left(S_{2ref}S_{3ref}\right)}{\left(\left(K_{2}K_{1}/S_{2ref}S_{3ref}\right)\left(1/h_{1}\right)\left(h_{1}+1+\left(K_{a}/K_{b}\right)h_{1}^{2}+\left(\left(A\beta \right)/{A\beta }_{ref}\right)h_{1}K_{i}\left({A\beta }_{ref}\right)\right)+\left(K_{1}/S_{2ref}S_{3ref}\right)\left(AcCoA\right)+\left(Ch\left(AcCoA\right)/S_{2ref}S_{3ref}\right)+\left(Ch/K_{3}\right)\left(1/S_{2ref}S_{3ref}\right)\right)}\\ X_{2}=\frac{E_{t}s_{31}s_{21}}{\left(\theta_{2}/h_{1}\left(1+h_{1}+\left(A\beta \right)h_{1}K_{i1}+\delta h_{1}^{2}\right)+\theta_{3}s_{31}+\theta_{5}s_{31}s_{21}+\theta_{4}s_{21}\right)}\\ X_{2}=\frac{E_{t}s_{31}s_{21}}{\left(\theta_{2}/h_{1}\left(1+h_{1}\left(1+A\beta K_{i1}\right)+\delta h_{1}^{2}\right)+\theta_{3}s_{31}+\theta_{5}s_{31}s_{21}+\theta_{4}s_{21}\right)}. \end{array}$$

Since *r*_1_ = *K*_2_′X_2_, therefore

(12) $$\begin{array}{c} r_{1}=\frac{\theta_{1}s_{31}s_{21}}{\theta_{2}/h_{1}\left(1+h_{1}\left(1+A\beta K_{i1}\right)+\delta h_{1}^{2}\right)+\theta_{3}s_{31}+\theta_{4}s_{21}+\theta_{5}s_{31}s_{21}}, \end{array}$$

where A*β* is the generated *β*-amyloid aggregates concentration in the dimensionless form and *K*_*i*_ is the dimensionless equilibrium rate constant for the first proposed inhibition mechanism (Figure 3(a)). Table 1 summarizes the nine ordinary-differential equations of 2E2C model considering ChAT-inhibition effect by *β*-amyloid based on the previous kinetic mechanism (1), and Table 2 gives the values of kinetic parameters.

#### 3.1.1. *β*-Amyloid Generation

**Figure 4:**
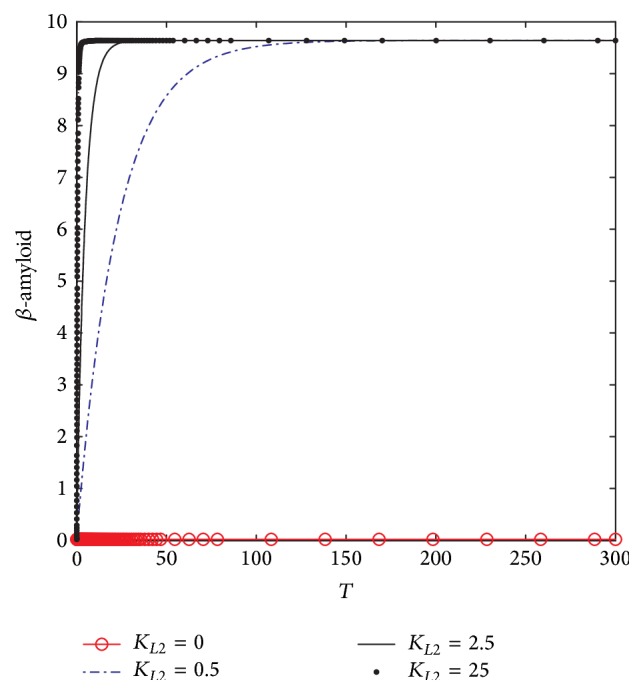
Time course of *β*-amyloid at different *K*_*L*2_ according to kinetic mechanism 1.

Figure 4 shows the dynamic behavior of *β*-amyloid aggregates concentrations. It is observed that, at *K*_*L*2_ = 0, no *β*-amyloid aggregate is produced. However, when *K*_*L*2_ increases to 0.5 (corresponding to 10 nM), the concentration of generated *β*-amyloid increases in the period 0–150 (corresponding to 0–15 hr) to reach the plateau at 9.75 (corresponding to 185 nM). However, when *K*_*L*2_ = 2.5 (corresponding to 50 nM), the produced *β*-amyloid increases rapidly to reach the plateau at the previous concentrations which is 185 nM but through a shorter time which is 15 (corresponding to 1.5 hr) and remains constant until the end of the incubation period which is 30 (corresponding to 30 hr). When *K*_*L*2_ increases to reach the maximum value which is 25 (corresponding to 500 nM), the generated A*β* increases with a rate faster than the previous rates at *K*_*L*2_ = 0.5 and 0.75 to reach the plateau in a period of 5 (corresponding to 0.5 hr only).

#### 3.1.2. Rate of ACh Synthesis and Hydrolysis

**Figure 5:**
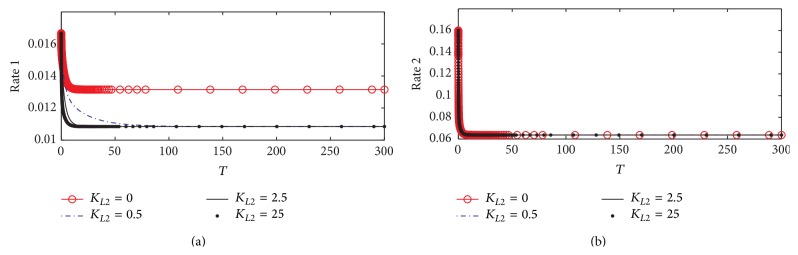
Time course according to kinetic mechanism 1 of (a) rate of ACh synthesis (Rate 1) and (b) rate of ACh hydrolysis (Rate 2) at different *K*_*L*2_.

Figure 5(a) shows the dynamics behavior of ACh synthesis (Rate 1) catalyzed by the enzyme ChAT. It is observed that Rate 1 reaches steady state at a high value which is 0.01375 at *K*_*L*2_ = 0, where no *β*-amyloid aggregate is produced; however, when *K*_*L*2_ increases to 0.5, 2.5, and 25, Rate 1 decreases significantly to reach a steady state around 0.0104. It is observed that the final value of Rate 1 at the end of the period 0–300 corresponding to 0–30 hr does not change with *K*_*L*2_ reflecting the neglected effect of the generated *β*-amyloid aggregates in this period. The rate of ACh synthesis is lowered by 25% through the effect of *β*-amyloid on ChAT.  Figure 5(b) shows that there is no effect of *K*_*L*2_ on the rate of hydrolysis (Rate 2) indicating that Rate 2 is independent of the change of *K*_*L*2_ in the kinetic mechanism.

These results are compatible with the experimental results obtained by Nunes-Tavares et al. [10] who showed that *β*-amyloid aggregates could bind to a significant portion of cholinergic neurons and reduce ChAT activity. It is observed that the reduction in Rate 1 decreases from 0.0105 to 0.000125 which refers to a reduction in ChAT activity of 99.7% with an increase in *K*_*L*2_ from 0 to 25 (corresponding to 500 nM). In addition, Nunes-Tavares et al. [10] showed that *β*-amyloid aggregates have a very limited effect on the activity of AChE in comparison to ChAT activity which is in agreement with our results shown in Figures 5(a) and 5(b).

#### 3.1.3. ACh Concentrations in Compartments 1 and 2

**Figure 6:**
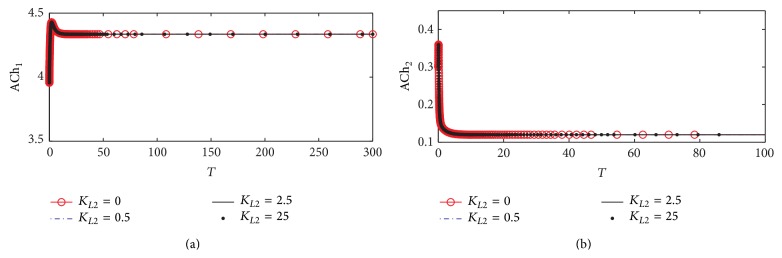
Time course according to kinetic mechanism 1 of (a) ACh concentration in compartment 1 (ACh_1_) and (b) ACh concentration in compartment 2 (ACh_2_) at different *K*_*L*2_ values.

One of the main physiological symptoms of AD is a reduction of ACh production in cholinergic neurons [38]. Therefore, the first step towards validating *β*-amyloid aggregates inhibition model is to see whether this physiological effect is reflected in the simulation results or not. As shown in Figure 6, it is an evidence of decreasing ACh concentration (ACh_1_) level in compartment 1.  Figure 6(a) shows the dynamics of ACh_1_ which reaches steady state at a high value at *K*_*L*2_ = 0 where ACh_1_ settles down around 4 (corresponding to 221.5 *µ*M) where no *β*-amyloid aggregate is produced, but when *K*_*L*2_ increases to 0.5, 2.5, and 25, it is observed that ACh_1_ decreases slightly to reach the steady state at 4.28 (corresponding to 215 *µ*M). It is noted that the final value of rate at the end of the period 0–300 corresponding to 0–30 hr does not change with *K*_*L*2_ reflecting the neglected effect of the generated *β*-amyloid aggregates in this period.

These results are compatible with experimental results obtained by Kar et al. [39] who indicated that the decrease in ChAT activity leads to a reduction in ACh generated in the presynaptic neurons. Furthermore, Zheng et al. [40] indicated that exposure of culture neurons to *β*-amyloid aggregates leads to a decrease in ChAT activity. However, Pedersen and Blusztajn [20] showed that only acetyl-coA not ChAT activity is affected negatively when exposed to *β*-amyloid aggregates leading to reduction of generated ACh similar to the rate of ACh hydrolysis shown in Figure 5(b) which exhibits no effect of *K*_*L*2_ where ACh_2_ does not change with any change in *K*_*L*2_. The absence of any observable effects in ACh_2_ is mostly likely due to the relative magnitude of the ACh synthesis rate (*r*_1_) compared to the magnitude of other transport phenomena within the model. Focusing on the kinetics in compartment 2, ACh_2_ level is governed mainly by two processes: membrane diffusion of ACh from compartment 1 and the rate of ACh degradation (*r*_2_) in compartment 2. The transport of ACh into compartment 2 through membrane diffusion is observed to slightly decrease due to reduced ACh_1_ levels from *β*-amyloid aggregates inhibition. On the other hand, the rate of ACh breakdown is maintained the same. It is expected that the influx of ACh into compartment 2 is approximately an order of magnitude higher than the consumption of ACh (*r*_2_) in that compartment. This means that although the influx of ACh into compartment 2 is limited by *β*-amyloid aggregates inhibition, the fact that *r*_2_ is so small in comparison infers that the overall level of ACh_2_ will not be affected much.

#### 3.1.4. Choline Concentrations in Compartments 1 and 2

**Figure 7:**
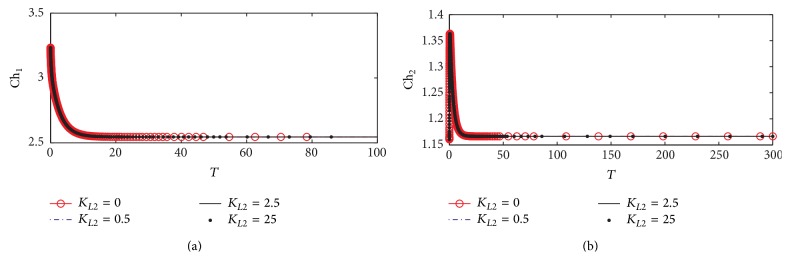
Time course according to kinetic mechanism 1 of (a) choline concentration in compartment 1 (Ch_1_) and (b) choline concentration in compartment 2 (Ch_2_) at different *K*_*L*2_ values.

Figures 7(a) and 7(b) show the dynamic response for choline concentrations in compartments 1 (Ch_1_) and 2 (Ch_2_), respectively. It is observed that there is no effect of *K*_*L*2_ on the behavior of Ch_1_. This reflects that the uptake of choline to compartment 1 is not affected by any change in *β*-amyloid aggregates through the interaction between ChAT and *β*-amyloid aggregates via the relevant kinetic mechanism. This result is compatible with the experimental results obtained by Nunes-Tavares et al. [10] who showed that there is no effect of *β*-amyloid oligomers on choline uptake.  Figure 7(b) shows that *K*_*L*2_ does not affect Ch_2_. In other words ChAT inhibition by *β*-amyloid aggregates in compartment 1 does not affect AChE activity in compartment 2.

#### 3.1.5. Acetate Concentrations in Compartments 1 and 2

**Figure 8:**
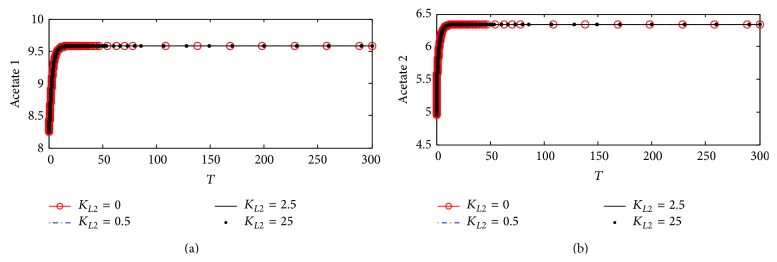
Time course according to kinetic mechanism 1 of (a) acetate concentration in compartment 1 (Ac_1_) and (b) acetate concentration in compartment 2 (Ac_2_) at different *K*_*L*2_ values.

Figure 8 shows the dynamic response for acetate concentrations in compartments 1 (Ac_1_) and 2 (Ac_2_).  Figure 8(a) shows that Ac_1_ increases from 8.25 at the beginning of the period and then it reaches the steady state value which is 9.51 (corresponding to 9.51 *µ*M) when *K*_*L*2_ increases to 0.5, 0.75, and 1. Ac_1_ increases slightly due to ChAT inhibition and accumulates due to low consumption of acetates by ChAT for synthesizing ACh. However, *K*_*L*2_ does not affect Ac_1_ since the steady state behavior of Ac_1_ is obtained when *K*_*L*2_ is higher than zero.  Figure 8(b) shows that there is no influence of *K*_*L*2_ on Ac_2_ which is the same as Ch_2_ and Rate 2 reflecting the negligible effect of *K*_*L*2_ on compartment 2 through the proposed kinetic mechanism 1.

#### 3.1.6. pH in Compartments 1 and 2

**Figure 9:**
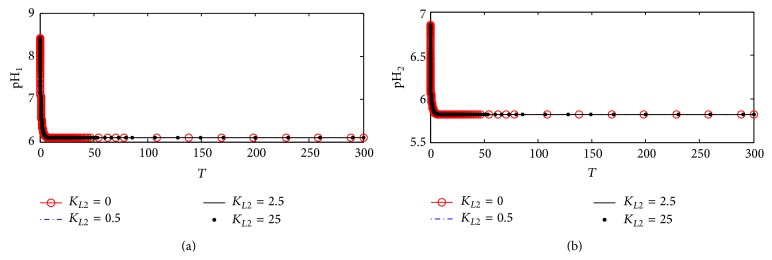
Time course according to kinetic mechanism 1 of (a) pH in compartment 1 (pH_1_) and (b) pH in compartment 2 (pH_2_) at different *K*_*L*2_ values.

Figure 9 shows the dynamic response of pH in both compartments 1 (pH_1_) and 2 (pH_2_).  Figure 9(a) shows that pH_1_ is affected very slightly with changes in *K*_*L*2_ indicating that the inhibition of ChAT activity with the proposed kinetic mechanism 1 has a low effect on changing pH_1_.  Figure 8(b) shows a negligible effect of *K*_*L*2_ on pH_2_ which is the same as the behaviors of Ch_2_ and Rate 2 explained previously. According to kinetic mechanism 1, *β*-amyloid aggregates promote a major reduction in ChAT activity while all other components in ACh cholinergic system remain unaffected.

The effect of *β*-amyloid aggregates inhibition on the remaining variables (pH and Ac) is observed to be negligible within the time frame studied. One likely explanation is that the feed concentrations of these chemical components into the 2E2C system are all much larger in magnitude compared to that of ACh. While they all have indirect dependence on the activity of ChAT through changes in ACh_1_ concentration, the reduction in ACh is too small to produce a significant effect on the concentration of these components.

## 4. Kinetic Mechanism 2: Competitive Inhibition of ***β***-Amyloid Aggregates with Enzyme Intermediate Complex X_2_

### 4.1. Formulation of Kinetic Mechanism 2

In this kinetic mechanism, there is a competitive inhibition of *β*-amyloid aggregates with enzyme intermediate complex X_2_. During ACh synthesis as shown in Figure 3(b), the modified rate equation can be derived as shown below.

X_1_ and X_2_ are related through the following expressions:

(13) $$\begin{array}{c} X_{1}\left(Ch\right)k_{1}=X_{2}k_{-1}\\ \text{or}\;\;X_{1}=\frac{K_{1}}{Ch}X_{2}, \end{array}$$

where *K*_1_ = *k*_−1_/*k*_1_.

By the assumption of rapid equilibrium, E_2_H and X_1_ can be related by

(14) $$\begin{array}{c} k_{2}\left(AcCoA\right)E_{2}H=k_{-2}X_{1}\\ \text{or}\;\;E_{2}H=\frac{K_{2}X_{1}}{\left(AcCoA\right)}=\frac{K_{2}K_{1}}{\left(AcCoA\right)\left(Ch\right)}X_{2}, \end{array}$$

where *K*_2_ = *k*_−2_/*k*_2_.

From the ionization of E_2_H,

(15) $$\begin{array}{c} E_{2}H\left(H^{+}\right)k_{-b}=E_{2}H^{+}k_{b}\\ \text{or}\;\;E_{2}H^{+}=\frac{\left(E_{2}H\right)\left(H^{+}\right)}{K_{b}}. \end{array}$$

From the deionization of E_2_H,

(16) $$\begin{array}{c} E_{2}^{-}\left({H_{}}^{+}\right)k_{a}=E_{2}Hk_{-a}\\ \text{or}\;\;E_{2}^{-}=\frac{K_{a}\left(E_{2}H\right)}{\left(H^{+}\right)}. \end{array}$$

From (16), (17), and (18), we get

(17) $$\begin{array}{c} E_{t2}=E_{2}H+{E_{2}}^{-}+E_{2}{H_{}}^{+}=\frac{K_{2}K_{1}}{\left(AcCoA\right)\left(Ch\right)}\left(1+\frac{K_{a}}{{H_{}}^{+}}+\frac{{H_{}}^{+}}{K_{b}}\right)X_{2}. \end{array}$$

X_2_ and X_3_ are related by

(18) $$\begin{array}{c} X_{3}=\frac{X_{2}}{K_{3}\left(AcCoA\right)}, \end{array}$$

where *K*_3_ = *k*_−3_/*k*_3_.

Also,

(19) $$\begin{array}{c} k_{i}{X_{2}}_{2}\left[A\beta \right]=k_{-i}X_{2}\left[A\beta \right]\\ \text{or}\;\;\;X_{2}\left[A\beta \right]=K_{i}{X_{2}}_{2}\left[A\beta \right], \end{array}$$

where *K*_*i*_ = *k*_*i*_/*k*_−*i*_.

Since E_*t*_ = E_*t*2_ + X_1_ + X_2_ + X_3_ + X_2_[A*β*], we get

(20) $$\begin{array}{c} E_{t}=X_{2}\left(\frac{K_{2}K_{1}}{AcCoA\left(Ch\right)}\left(1+\frac{K_{a}}{{H_{1}}^{+}}+\frac{{H_{1}}^{+}}{K_{b}}\right)+\frac{K_{1}}{Ch}+1+\frac{1}{K_{3}\left(AcCoA\right)}+\left(A\beta \right)K_{i}\right)\\ \text{or}\;\;X_{2}=\frac{E_{t}\left(Ch\right)\left(AcCoA\right)}{\left(K_{2}K_{1}\left(1+\left(K_{a}/{H_{1}}^{+}\right)+\left({H_{1}}^{+}/K_{b}\right)\right)+K_{1}\left(AcCoA\right)+Ch\left(AcCoA\right)+\left(Ch/K_{3}\right)+\left(A\beta \right)K_{i}Ch\left(AcCoA\right)\right)}. \end{array}$$

Since *R*_1_ = *K*_2_′X_2_, therefore,

(21) $$\begin{array}{c} r_{1}=\frac{\theta_{1}s_{21}s_{31}}{\left(\theta_{2}/h_{1}\left(h_{1}+1+\delta h_{1}^{2}\right)+\theta_{3}s_{31}+\theta_{4}s_{21}+\theta_{5}s_{21}s_{31}\left(1+K_{I1}A\beta \right)\right)}, \end{array}$$

where A*β* is the concentration of generated *β*-amyloid aggregates in the dimensionless form and *K*_*I*1_ is the dimensionless equilibrium rate constant for the second proposed inhibition mechanism shown in Figure 3(b). In the next section, the results presented are based on the previous equation of *r*_1_ replacing the *r*_1_ in Table 1 while all other equations remain the same. The kinetic parameter values are given in Table 2.

### 4.2. Results of Kinetic Mechanism 2

Incorporating the rate of ChAT synthesis *r*_1_ (21) inhibited by *β*-amyloid aggregates derived from the kinetic mechanism (2) into the 9th dimension 2E2C model shown in Table 1, the effect of *β*-amyloid aggregates on ACh neurocycle is investigated to check the feasibility of kinetic mechanisms as a descriptor for the interaction between ChAT activity and *β*-amyloid aggregates.

#### 4.2.1. *β*-Amyloid Generation

**Figure 10:**
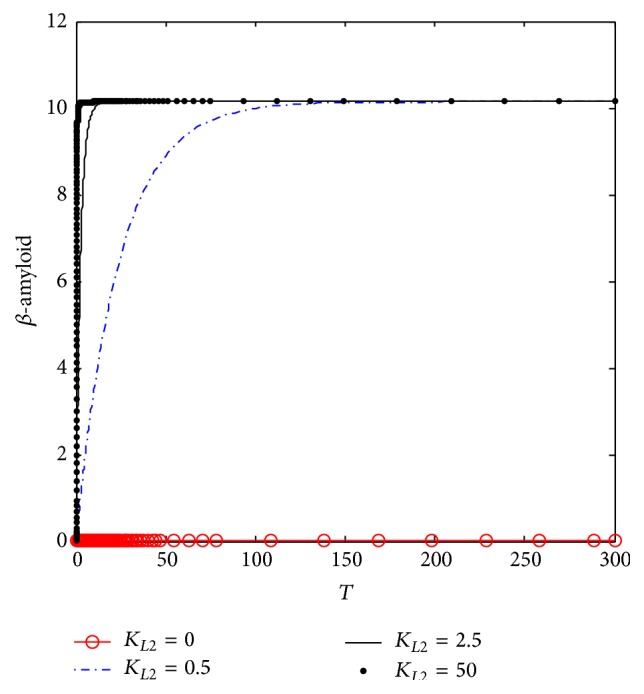
Time course of generated *β*-amyloid aggregates at different *K*_*L*2_ (according to kinetic mechanism 2).

Figure 10 shows the dynamic behavior of *β*-amyloid aggregates produced in compartment 1. It is clear that at *K*_*L*2_ = 0, where there is no inlet *β*-amyloid, there is no generation of *β*-amyloid aggregates. However, when *K*_*L*2_ increases to 0.5 (corresponding to 10 nM), *β*-amyloid aggregate is produced to reach 10 (corresponding to 200 nM) after a time of 150 (corresponding to 15 hr); then it becomes constant where there is no more generation until the end of the incubation period (300) corresponding to 30 hr. In addition, Figure 10 shows that when *K*_*L*2_ increases to 2.5 (corresponding to 50 nM), *β*-amyloid aggregates are generated faster than that at *K*_*L*2_ = 0.5, where *β*-amyloid aggregates reach the stationery steady state after 15 (corresponding to 1.5 hr); then it does not change until the end of the incubation period. Furthermore, at *K*_*L*2_ = 25 (corresponding to 500 nM), *β*-amyloid aggregates are produced much faster than before where it reaches the steady state value only after a dimensionless time of 7 (corresponding to 42 minutes). Comparing the dynamic behaviors of *β*-amyloid aggregates in kinetic mechanism 2 and kinetic mechanism 1, it is observed that the final steady state value of kinetic mechanism 2 is higher than that of kinetic mechanism 1. The increase of final *β*-amyloid aggregates might lead to changes in other state variables of the system.

#### 4.2.2. Rates of ACh Synthesis and Hydrolysis

**Figure 11:**
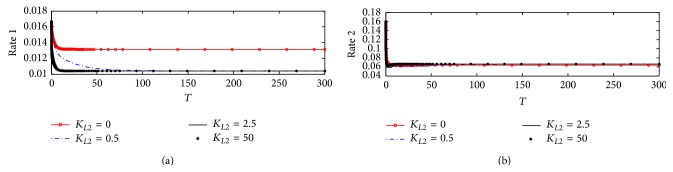
Time course of (a) rate of ACh synthesis (Rate 1) and (b) rate of ACh hydrolysis (Rate 2) at different *K*_*L*2_ (according to kinetic mechanism 2).

Figure 11 shows the dynamic behavior of rate of ACh synthesis catalyzed by ChAT (Rate 1) and rate of ACh hydrolysis catalyzed by AChE (Rate 2).  Figure 11(a) shows that Rate 1, at no *β*-amyloid aggregates production, where *K*_*L*2_ = 0, decreases from 0.016 at the beginning of process to reach 0.0138 after passing 20 (corresponding to 2 hr); then it becomes constant until the end of the period. However, when ChAT activity is reduced according to kinetic mechanism 2 at *K*_*L*2_ = 0.5 (corresponding to 10 nM), Rate 1 decreases significantly as shown in Figure 11(a) to reach 0.0103 at the end of the period but it reaches the steady state slower than that at *K*_*L*2_ = 0 where it takes time of 100 (corresponding to 10 hr) to reach the plateau. Furthermore, when *K*_*L*2_ increases to 2.5 and 50 (corresponding to 50 and 1000 nM, resp.), the generated *β*-amyloid takes the same behavior where both decrease with the same rate but faster than that at *K*_*L*2_ = 0.5. It is observed that the reduction in Rate 1 decreases from 0.01375 to 0.0105 which refers to a reduction in ChAT activity of 24.7% with an increase of *K*_*L*2_ from 0 to 25 (corresponding to 500 nM).  Figure 11(b) shows that the effect of changing *K*_*L*2_ on Rate 2 is negligible according to the kinetic mechanism 2 indicating the same behavior as kinetic mechanism 1 as shown previously in Figure 7(b).

#### 4.2.3. ACh Concentrations in Compartments 1 and 2

**Figure 12:**
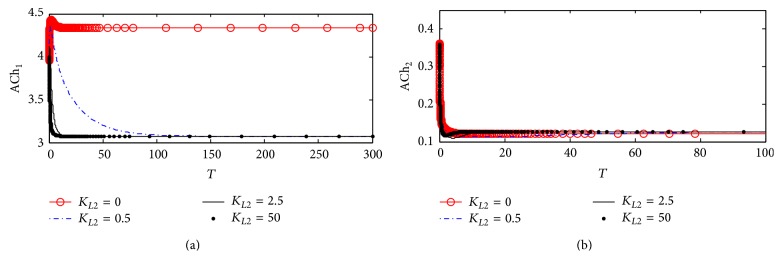
Time course of (a) ACh concentration in compartment 1 (ACh_1_) and (b) ACh concentration in compartment 2 (ACh_2_) at different *K*_*L*2_ values (according to kinetic mechanism 2).

Figure 12(a) shows the dynamics of ACh_1_ with the rate of ChAT activity inhibition *r*_1_ based on kinetic mechanism 2. It is observed that at *K*_*L*2_ = 0.5 ACh_1_ reduces from 4.3 (corresponding to 216.419 *µ*M) at the beginning of *β*-amyloid aggregates incubation to reach 3.15 (corresponding to 158.5 *µ*M) after passing 100 (10 hr) and it keeps at this value until the end of the period which is 300 (corresponding to 30 hr). Comparing ACh_1_ at *K*_*L*2_ = 0.5 to that in the first kinetic mechanism in Figure 6(a), it is observed that the rate of ChAT activity affected by the interaction between competitive inhibitions of *β*-amyloid aggregates with enzyme intermediate complex X_2_ in kinetic mechanism 2 leads to a more reduction in ACh_1_. At *K*_*L*2_ = 2.5 and 50, the reduction of ACh_1_ is faster than that at *K*_*L*2_ = 0.5 where it occurs after passing 15 and 10 (corresponding to 1.5 and 1 hr, resp.); then ACh_1_ remains at steady state around 3.15 (corresponding to 158.5 *µ*M) indicating that inhibition of ChAT activity by *β*-amyloid aggregates reached its maximum limit.  Figure 12(b) shows that ACh_2_ is not affected significantly with the change of *K*_*L*2_, which is the same as in kinetic mechanism 1 as shown in Figure 6(b).

#### 4.2.4. Choline Concentrations in Compartments 1 and 2

**Figure 13:**
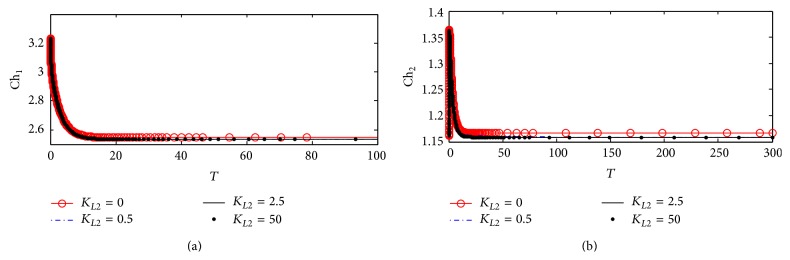
Time course of (a) choline concentration in compartment 1 (Ch_1_) and (b) choline concentration in compartment 2 (Ch_2_) at different *K*_*L*2_ values (according to kinetic mechanism 2).

Figures 13(a) and 13(b) illustrate the time course of choline concentrations in compartments 1 and 2, respectively. It is observed that *K*_*L*2_ has no effect on both Ch_1_ and Ch_2_ indicating that the inhibition of ChAT activity by *β*-amyloid aggregate does not affect choline uptake in compartment 1 and choline produced from hydrolysis in compartment 2 following the same behavior in kinetic mechanism 1 as shown previously in Figures 7(a) and 7(b).

#### 4.2.5. Acetate Concentrations in Compartments 1 and 2

**Figure 14:**
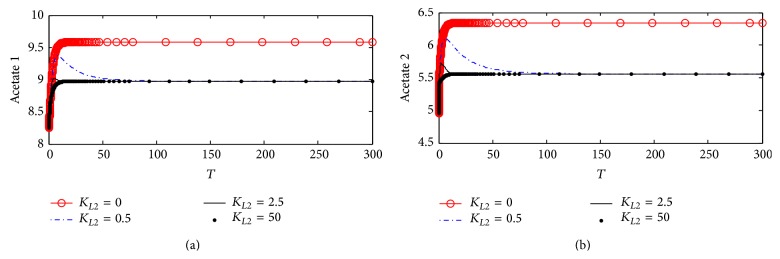
Time course according to kinetic mechanism 2 of (a) acetate concentration in compartment 1 (Ac_1_) and (b) acetate concentration in compartment 2 (Ac_2_) at different *K*_*L*2_ values.

Figure 14(a) shows the effect of changing *K*_*L*2_ in terms of the inhibition of ChAT activity according to kinetic mechanisms 2 on intracellular concentration of acetate. At *K*_*L*2_ = 0, Ac_1_ increases through the first 30 (3 hr) and reaches steady state around 9.52 (corresponding to 9.52 *µ*M) indicating that inhibition of ChAT activity by *β*-amyloid aggregates reached its maximum limit. However, after incorporating *β*-amyloid generation in terms of *K*_*L*2_ = 0.5, 2.5, and 50 (corresponding to 10, 25, 1000 nM), there is a significant decrease in Ac_1_, where the final steady state concentration of Ac_1_ is around 8.95 (corresponding to 8.95 *µ*M). It is clear that competitive inhibition of *β*-amyloid aggregates with enzyme intermediate complex X_2_ in kinetic mechanism 2 has more effect on Ac_1_ than that in kinetic mechanism 1.  Figure 14(b) shows that Ac_2_ is affected clearly with any increase in inlet *β*-amyloid aggregates concentrations (*K*_*L*2_) where, at *K*_*L*2_ = 0, the steady state of Ac_2_ = 6.4 (corresponding to 6.4 *µ*M) while with the increase of *K*_*L*2_ to 0.5, 2.5, and 50 the steady state value of Ac_2_ will be around 5.6 and not varying with changing *K*_*L*2_ as illustrated in Figure 14(b). This indicates again that inhibition of ChAT activity by *β*-amyloid aggregate reaches the maximum effect.

#### 4.2.6. pH in Compartments 1 and 2

**Figure 15:**
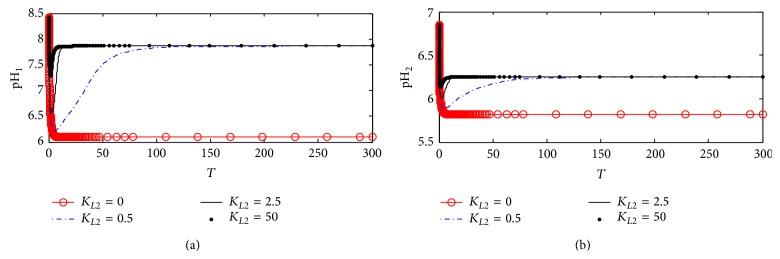
Time course according to kinetic mechanism 2 of (a) pH in compartment 1 (pH_1_) and (b) pH in compartment 2 (pH_2_) at different *K*_*L*2_ values.

Figure 15(a) shows the dynamics of pH_1_ with changing *K*_*L*2_. It is observed that, at *K*_*L*2_ = 0, pH_1_ decreases from 8.5 at the beginning to settle down around 6.2 at the end of the period. However, as *K*_*L*2_ increases to 0.5, 2.5, and 50, pH_1_ decreases clearly to settle down around 7.75 indicating that the inhibition of ChAT activity by *β*-amyloid reaches the maximum limit. It is concluded that the rate of transport of hydrogen protons from compartment 1 to compartment 2 is still higher than the rate of accumulation of hydrogen protons due to the inhibition of ChAT catalytic effect according to kinetic mechanism 2.  Figure 15(b) shows the dynamic behavior of pH_2_ where pH_2_ increases from 5.85 at *K*_*L*2_ = 0 to the steady state value of 6.23 with increasing *K*_*L*2_ from 0.5 to 50.

It is clear that kinetic mechanism 2 in terms of competitive inhibition of *β*-amyloid aggregates with enzyme intermediate complex X_2_ has more effect on pH_1_ and pH_2_ than that in kinetic mechanism 1. It is observed that, after time of 100 (corresponding to 10 hr), there is no effect for any further change of *β*-amyloid aggregates production rate (*K*_*L*2_ from 0.5 to 25), where all state variables reach the same steady state values indicating that the activity of ChAT is fully inhibited. These results are compatible with that obtained by Nunes-Tavares et al. [10] who showed that there is no significant effect for any further *β*-amyloid concentration more than 100 nM. According to kinetic mechanism 2, *β*-amyloid aggregates promote a major reduction in ChAT function, ACh_1_, acetate 1 and acetate 2, pH_1_, and pH_2_ while all other components in ACh cholinergic system remain unaffected.

## 5. Kinetic Mechanism 3: Noncompetitive Inhibition of ***β***-Amyloid Aggregates with All Species ChAT 

### 5.1. Formulation of Kinetic Mechanism 3

**Figure 16:**
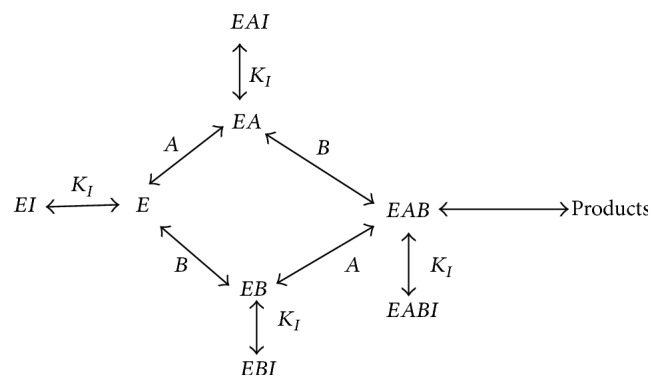
Possible noncompetitive inhibition mechanisms for *β*-amyloid aggregates during pH-dependent ChAT synthesis of ACh: E = ChAT, I = *β*-amyloid aggregates, A = choline, B = acetyl-coA, and Products = ACh.

*β*-amyloid aggregate inhibitor behaves as a noncompetitive inhibitor and could attack all ChAT species in the synthesis reaction in compartment 1 with the same affinity if the reaction follows either a rapid equilibrium random mechanism or an ordered sequential [41].  Figure 16 shows the kinetic mechanism 3 for the synthesis reaction catalyzed by ChAT where all species in ChAT can be exposed to *β*-amyloid aggregates, where E = ChAT, I = *β*-amyloid aggregates, A = choline, and B = acetyl-coA. In the same way as the previous kinetic mechanisms 1 and 2 were derived, the final form of the rate of ACh synthesis derived is shown as follows:

(22) $$\begin{array}{c} r_{1}=\frac{\theta_{1}s_{21}s_{31}}{\left(\theta_{2}/h_{1}\left(h_{1}+1+\delta h_{1}^{2}\right)+\theta_{3}s_{31}+\theta_{4}s_{21}+\theta_{5}s_{21}s_{31}\right)\left(1+K_{I1}A\beta \right)}. \end{array}$$

By using (22) for the rate equation *r*_1_ in 2E2C of Table 1 and keeping all other equations the same and incorporating *K*_*I*1_ into Table 2, the effect of *β*-amyloid aggregates as an inhibitor for ChAT activity on 2E2C model can be simulated as discussed in the next section.

### 5.2. Results of Kinetic Mechanism 3

#### 5.2.1. *β*-Amyloid Aggregates Generation

**Figure 17:**
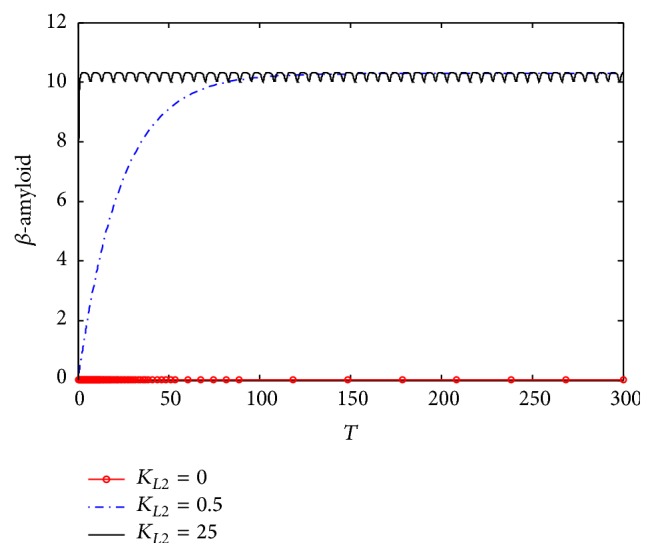
Time course of *β*-amyloid at different *K*_*L*2_ (according to kinetic mechanism 3).

Figure 17 shows the dynamic response at different *K*_*L*2_ values. At *K*_*L*2_ = 0 where there is a normal ChAT activity without inhibition, it is observed that there is no *β*-amyloid aggregates generated where *β*-amyloid concentration = 0. At *K*_*L*2_ = 0.5, *β*-amyloid aggregate is produced significantly and increases to 10.2 (corresponding to 204 nM) after passing time of 100 (corresponding to 10 hr), where it reaches steady state at this value. At *K*_*L*2_ = 25 (corresponding to 500 nM), it is observed that *β*-amyloid aggregate oscillates in a narrow range (10–10.2) corresponding to (200–204 nM). The oscillatory behavior is an interesting phenomenon because it may affect other state variables of the system as will be investigated below.

#### 5.2.2. Rate of ACh Synthesis and Hydrolysis

**Figure 18:**
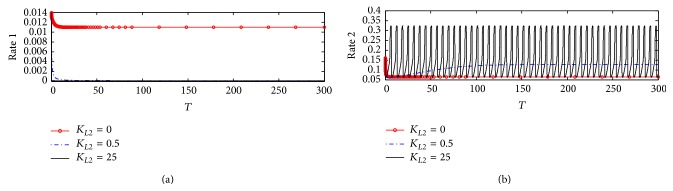
Time course of (a) rate of ACh synthesis (Rate 1) and (b) rate of ACh hydrolysis (Rate 2) at different *K*_*L*2_ (according to kinetic mechanism 3).

Figure 18(a) shows the effect of varying inlet *β*-amyloid *K*_*L*2_ on ACh synthesis Rate 1 under the condition of ChAT fully inhibited according to (21). At normal ChAT synthesis where there is no *β*-amyloid aggregates generation, Rate 1 decreases from 0.014 at the beginning to 0.0105 which is the steady state value after passing time of 200 (corresponding to 20 hr). However, when *K*_*L*2_ increases to 0.5 and 25 (corresponding to 10 and 500 nM), Rate 1 decreases from 0.014 at the beginning of *β*-amyloid aggregates incubation to reach a very small value close to zero indicating that ChAT activity is completely inhibited by *β*-amyloid aggregates which attack every species in ChAT enzyme. It is observed that the reduction in Rate 1 decreases from 0.0105 to 0.000125 which refers to a reduction in ChAT activity of 99.7% with increasing *K*_*L*2_ from 0 to 25 (corresponding to 500 nM). This result is consistent with the experimental results obtained by Nunes-Tavares et al. [10] who showed that *β*-amyloid could inhibit ChAT completely.

Figure 18(b) shows that a very interesting phenomenon which is the oscillatory behavior at a high inlet *β*-amyloid aggregates *K*_*L*2_ = 25 where Rate 2 oscillates in the range (0.05–0.32) This oscillatory behavior reflects the disturbances occurring in the cholinergic system at which *β*-amyloid aggregates are generated at a high rate as shown in Figure 17 and acts severely as an inhibitor ChAT.

#### 5.2.3. ACh Concentrations in Compartments 1 and 2

**Figure 19:**
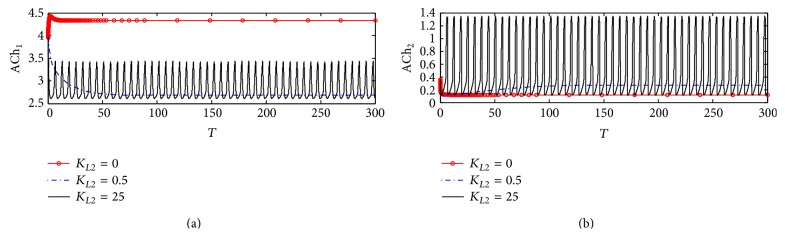
Time course of (a) ACh concentration in compartment 1 (ACh_1_) and (b) ACh concentration in compartment 2 (ACh_2_) at different *K*_*L*2_ values (according to kinetic mechanism 3).

Figure 19(a) shows that ACh_1_ is synthesized with a high rate at *K*_*L*2_ = 0 where ACh_1_ settles down to around 35 (corresponding to 219 *µ*M) at the end of the time *T* = 200 (corresponding to 30 hr). When *K*_*L*2_ = 0.5 (corresponding to 10 nM), the levels of ACh_1_ decrease to 2.7 (corresponding to 136 *µ*M) after passing *T* = 100 (corresponding to 10 hr); then ACh_1_ remains constant at the latter concentration until the end of the incubation period which is 30 hr. As *K*_*L*2_ increases to 25 (corresponding to 500 nM), the interesting oscillatory phenomenon appears where ACh_1_ oscillates in a wide range from 2.6 to 3.4 corresponding to 131–322 *µ*M. This range shows that the ACh_1_ moves in disturbance and could be related to irregular behavior leading to impaired memory.  Figure 19(b) shows that, at the highest value of *K*_*L*2_ = 25, ACh_2_ fluctuates in a wide range (0.1–1.38) corresponding to 5–69.5 *µ*M. The latter fluctuation shows the irregular behavior of ACh_2_ released in compartment 2 and could lead to unreasonable electrical and chemical messages to the postsynaptic receptors and finally dysfunctions of the memory. The latter disturbances show the harmful effect of high concentration *β*-amyloid aggregates and the consequences of inhibition action with all species in ChAT.

#### 5.2.4. Choline Concentrations in Compartments 1 and 2

**Figure 20:**
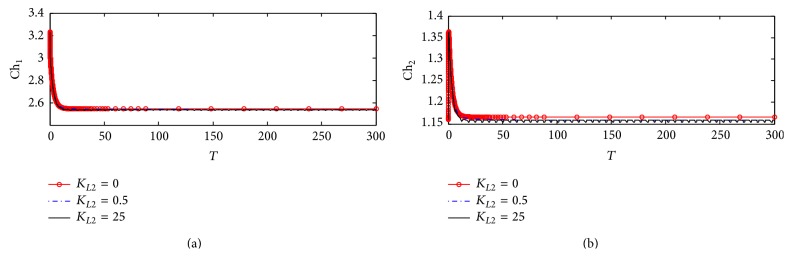
Time course of (a) choline concentration in compartment 1 (Ch_1_) and (b) choline concentration in compartment 2 (Ch_2_) at different *K*_*L*2_ values (according to kinetic mechanism 3).

**Figure 21:**
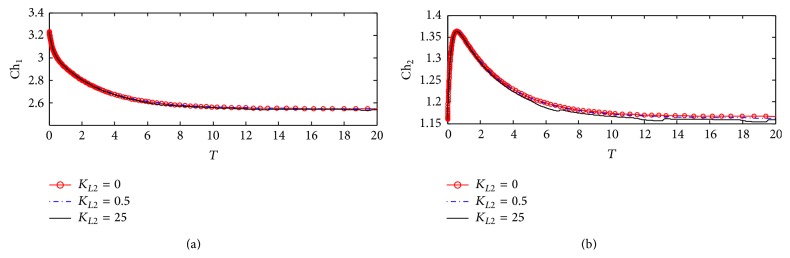
Magnification of Figure 20: (a) choline concentration in compartment 1 (Ch_1_) and (b) choline concentration in compartment 2 (Ch_2_) at different *K*_*L*2_ values (according to kinetic mechanism 3).

**Figure 22:**
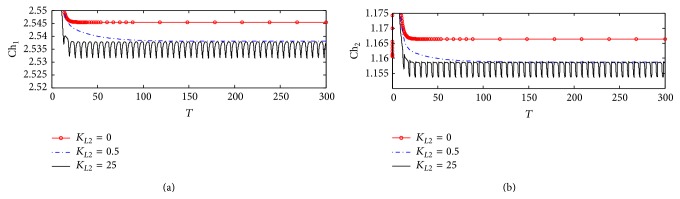
Magnification of Figure 20: (a) choline concentration in compartment 1 (Ch_1_) and (b) choline concentration in compartment 2 (Ch_2_) at different *K*_*L*2_ values (according to kinetic mechanism 3).

Figure 20(a) shows that there are no observable effects in the behavior of Ch_1_. This reflects that there is no effect of ChAT inhibition by *β*-amyloid aggregates on intracellular choline concentration indicating that both choline uptake and choline transport between compartments 1 and 2 are able to keep balanced intracellular choline concentration. It is observed that Ch_1_ decreases from 3.22 (corresponding to 322 *µ*M) at the beginning of incubation to 2.55 (corresponding to 255 *µ*M).  Figure 20(b) shows a limited effect of varying *K*_*L*2_ on Ch_2_. Ch_2_ increases very fast at the beginning of the reaction from 1.15 (corresponding to 115 *µ*M) to 1.36 (corresponding to 136 *µ*M) in just a short time *T* = 0–2 corresponding to 0–0.2 hr and then decreases to settle down at 1.16.  Figure 21(a) shows that Ch_1_ decreases from 3.2 (corresponding to 320 *µ*M) to 2.52 (corresponding to 252 *µ*M) after passing *T* = 20 (corresponding to 2 hr). Figures 22(a) and 22(b) are enlargement of Figures 20(a) and 20(b) where it is observed that Ch_1_ oscillates in a narrow range (2.532–2.538) (corresponding to 253.2–2538 *µ*M) at *K*_*L*2_ = 25 (corresponding to 500 nM) as shown in Figure 22(a).  Figure 22(b) shows that Ch_2_ oscillates through a limited range of 1.1548–1.1558 (corresponding to 115.48–155.8 *µ*M).

The limited effect of A*β* aggregates inhibition on Ch_1_ and Ch_2_ is found to be negligible within the time frame studied. One likely explanation is that the choline uptake and the rate of ACh hydrolysis are all much larger in magnitude compared to that of ACh. While they all have an indirect dependence on the activity of ChAT through changes in ACh_1_ concentration, the reduction in ACh is too small to produce a significant effect on the concentrations of these components.

#### 5.2.5. Acetate Concentrations in Compartments 1 and 2

**Figure 23:**
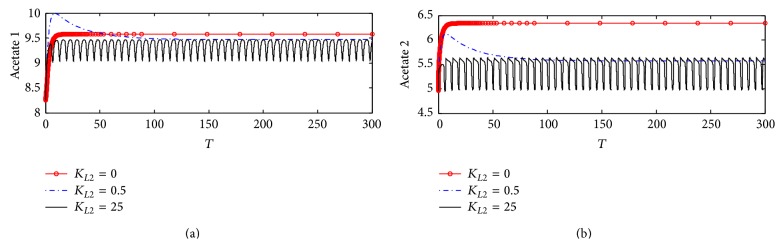
Time course of (a) acetate concentration in compartment 1 (Ac_1_) and (b) acetate concentration in compartment 2 (Ac_2_) at different *K*_*L*2_ values (according to kinetic mechanism 3).

Figure 23(a) shows the dynamic behaviors of acetate 1 at different *K*_*L*2_ values. It is observed that, at *K*_*L*2_ = 0 where no *β*-amyloid aggregates are produced, acetate 1 increases to settle down at the stationery state value of 9.6 (corresponding to 6.6 *µ*M). As *K*_*L*2_ increases to 0.5 (corresponding to 10 nM), acetate 1 settles down at a lower value of 9.45 (corresponding to 9.45 *µ*M). When *K*_*L*2_ increases to the highest value of 25 (corresponding to 500 nM), acetate 1 fluctuates in the range of 9–9.5 (corresponding to 9–9.5 *µ*M).

Figure 23(b) shows that acetate 2, at *K*_*L*2_ = 0, takes steady state behavior which is around 6.4 (corresponding to 6.4 *µ*M). While *K*_*L*2_ increases to 0.5 (corresponding to 10 nM), acetate 2 deceases to settle down around 5.6 (corresponding to 5.6 *µ*M) which is lower than that at no inhibition (*K*_*L*2_ = 0). When *K*_*L*2_ increases to 25 (corresponding to 500 nM), the oscillatory behavior dominates the system where acetate 2 fluctuates in the range 5–5.63 corresponding to 5–5.63 *µ*M.

#### 5.2.6. pH in Compartments 1 and 2

**Figure 24:**
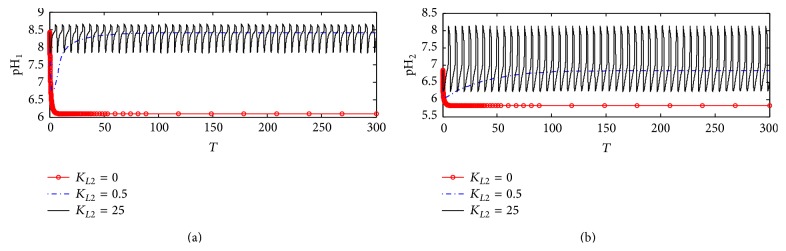
Time course of (a) pH in compartment 1 (pH_1_) and (b) pH in compartment 2 (pH_2_) at different *K*_*L*2_ values (according to kinetic mechanism 3).

Figure 24(a) shows the dynamic response of pH_1_ at different *K*_*L*2_ values. It is observed that, at *K*_*L*2_ = 0 where no *β*-amyloid aggregate is produced, pH_1_ decreases rapidly from 8.5 to settle down at 6.2. At *K*_*L*2_ = 0.5, pH_1_ decreases from 8.5 to 6.75 in the period 0–5 corresponding to 0–0.5 hr; then it increases to settle down around 8.4 which is the steady state value. When *K*_*L*2_ increases to the high value of 25 (corresponding to 500 nM), pH_1_ oscillates in the range of 7.8–8.6.

Figure 24(b) shows that, at *K*_*L*2_ = 0, pH_2_ settles down around 5.8; when *K*_*L*2_ increases to 0.5 (corresponding to 10 nM), pH_2_ decreases from 6.65 to 6.1 in the period 0–5 corresponding to 0–0.5 hr; then it increases to settle down around the steady state value of 6.8. It is observed that, at *K*_*L*2_ = 25, pH_2_ fluctuates in a wide range of 6.35–8.2 causing disturbances to the cholinergic system.

## 6. Discussion

The effect of *β*-amyloid aggregates inhibition on ChAT activity is investigated through proposing three different kinetic mechanisms. The first kinetic mechanism is that *β*-amyloid aggregate binds to active enzyme complex E_2_H in ChAT for producing ACh while in the second kinetic mechanism *β*-amyloid aggregates competitively bind to enzyme intermediate X_2_. In the third kinetic mechanism, *β*-amyloid aggregate binds all species in ChAT enzyme. In each kinetic mechanism, a rate equation of ACh synthesis (*r*_1_) is derived and incorporated into the two-enzyme/two-compartment model to get a cholinergic system with nine first-order ordinary differential equations. The concentrations of all state variables in compartments 1 and 2 in addition to the rates of ACh synthesis and ACh hydrolysis are investigated at different rates of *β*-amyloid aggregates generation (*K*_*L*2_) and compared with normal cholinergic neurons where there is no inhibition with *β*-amyloid aggregates.

It is found that for all kinetic mechanisms the concentration of generated *β*-amyloid aggregates increases with increasing *β*-amyloid aggregates production until it reaches saturation levels where there is no further increase with any additional increase of *K*_*L*2_. In the first kinetic mechanism where *β*-amyloid aggregate attacks only the active enzyme complex E_2_H in ChAT, it is found that the rate of ACh synthesis and the concentration of intracellular ACh (ACh_1_) decrease significantly with an increase in *K*_*L*2_ referring to the inhibition effect of ChAT activity while all of the other rates of ACh hydrolysis are not affected. In addition, ACh concentration in compartment 2 (ACh_2_), choline concentrations in compartments, pH in both compartments, and acetate concentrations in both compartments are not affected by any change in *K*_*L*2_. Therefore, the interaction between *β*-amyloid aggregates and enzyme complex E_2_H in ChAT has a limited effect on most of elements of ACh neurocycle. One of the main explanations is that the limited effect of *β*-amyloid aggregates on substrates such as choline and acetyl-CoA in kinetic mechanism 1 is because of the idea that supply of these substrates such as choline from compartment 2 to compartment 1 and choline transport from compartment 1 to compartment 2 and acetate transport from compartment 1 to compartment 2 can compensate any reduction due to the inhibiting effect of *β*-amyloid aggregates on the active site E_2_H. However, the significant reduction in ACh concentrations due to ChAT activity inhibition is one of the hallmarks in cholinergic dysfunctions.

The results of the second kinetic mechanism where *β*-amyloid aggregates competitively attack the enzyme intermediate X_2_ are similar to that of the first kinetic mechanism except that acetate concentration in compartments 1 and 2 decreases significantly while pH_1_ and pH_2_ in both compartments increase with the increase in *K*_*L*2_ where the attack of *β*-amyloid aggregates on intermediate X_2_ of ChAT leads to a decrease in the consumption of acetyl-CoA in compartment 1 and the lower production of acetate in compartment 2. The reduction in Rate 1 or ChAT activity in kinetic mechanisms 1 and 2 decreases with around 25% with increase in *K*_*L*2_ from 0 to 25 (corresponding to 500 nM).

In the third kinetic mechanism, where *β*-amyloid aggregates bind to all species in ChAT, it is found that, at low values of *K*_*L*2_, the behavior of cholinergic ACh looks like that of the second kinetic mechanism. However, at high values of *K*_*L*2_ (e.g., 25), an interesting phenomenon arises for all state variables of the system where all variables fluctuate in a wide range (as shown from Figures 17 to 22) leading to irregular behavior of the cholinergic system. These disturbances occurring in the cholinergic system are one of the main features of AD [10]. According to kinetic mechanism 3, *β*-amyloid aggregates promote a major reduction in ChAT activity. Kar et al. [23] indicated that decline of intracellular ACh concentration is due to reduction in ChAT catalytic action. Moreover, it was observed that long incubation of rat primary septal culture neurons with micromolar concentration of *β*-amyloid aggregates caused a reduction in ChAT function and cell death [23, 42].

The results of kinetic mechanism 3 are compatible with the experimental results obtained by Nunes-Tavares et al. [10] who showed that *β*-amyloid aggregates could bind to a significant portion of cholinergic neurons and reduce ChAT activity completely. It is observed that the reduction in Rate 1 in kinetic mechanism 3 decreases from 0.0105 to 0.000125 which refers to a reduction in ChAT activity of 99.7% with increasing *K*_*L*2_ from 0 to 25 (corresponding to 500 nM). The results are in agreement with the experimental results obtained by Nunes-Tavares et al. [10] who showed that *β*-amyloid could inhibit ChAT completely.

It is observed that, after time of 100 (corresponding to 10 hr), there is no effect for any further change of *β*-amyloid aggregates production rate (*K*_*L*2_ from 0.5 to 25), where all state variables reach the same steady state values. These results are compatible with those obtained by Nunes-Tavares et al. [10] who showed that there is no significant effect for any further *β*-amyloid concentration higher than 100 nM. It is also observed that ChAT inhibition by *β*-amyloid could cause severe consequences for the rate of ACh synthesis and ACh concentrations in compartments 1 and 2 as well, where ChAT inhibition could reach 100% as shown in Figure 18(a).

## 7. Summary and Conclusions

In this work, the effect of *β*-amyloid aggregates as an inhibitor on ChAT activity for ACh synthesis through the two-enzyme/two-compartment (2E2C) model in a variety of situations is analyzed through suggesting three different kinetic mechanisms for the inhibition effect. Overall, numerical solutions to the modified 2E2C with *β*-amyloid aggregates were in accordance with three significant, widely reported symptoms of AD: loss of ChAT activity [20], reduced choline uptake [39], and reduced ACh production [20]. This in turn means that the direct inactivation of ChAT by *β*-amyloid aggregates may be a probable mechanism contributing to the development of AD. The incorporation of ChAT inhibition by *β*-amyloid aggregates into the 2E2C model is able to yield dynamic solutions for concentrations of generated *β*-amyloid aggregates, ACh, choline, acetate, and pH in addition to the rates of ACh synthesis and ACh hydrolysis in compartments 1 and 2. This correlates well with the physiological understanding since the production of *β*-amyloid is not generally known to be highly reversible. One of the most significant physiological symptoms of AD is the reduction of ACh neurotransmitter concentration within cholinergic neurons. In this investigation, the effect of ChAT activity inhibition via *β*-amyloid is considered an individual basis in order to evaluate the validity of each kinetic mechanism, since it is highly likely that physiologically more than one single mechanism can contribute to the generation of AD symptoms. In comparison to the effect of *β*-amyloid via choline leakage hypothesis made by Ehrenstein et al. [28], it is observed that ChAT activity needs a high concentration of *β*-amyloid production rate (*K*_*L*2_). This is in agreement with the experimental results obtained by Kar et al. [39] who showed that the exposure to small concentrations of *β*-amyloid aggregates has no impact on ChAT activity in cortex tissues. However, the results are in agreement with those obtained by Zambrzycka et al. [9] who showed that when neurons tissues are exposed to high concentrations of *β*-amyloid for a while, ChAT activity is reduced significantly. The results of kinetic mechanism 3 are in agreement with that obtained by Mustafa et al. [43, 44] who indicated the oscillatory behavior of ACh neurocycle at low ChAT activity.

*Dimensionless State Variables, Parameters, and Other Terms [5, 6]*

*Dimensionless State Variables*

 
*s*_1(*j*)_ = [*S*_1_]_(*j*)_/*K*_*s*__1_: dimensionless ACh concentration in compartment *j*.
 
*s*_2(*j*)_ = [*S*_2_]_(*j*)_/[*S*_2_]_ref_: dimensionless choline concentration in compartment *j*.
 
*s*_3(*j*)_ = [*S*_3_]_(*j*)_/[*S*_3_]_ref_: dimensionless acetate concentration in compartment *j*.
 
*h*_(*j*)_ = [H^+^]_(*j*)_/*K*_*h*__1_: dimensionless hydrogen ion concentration in compartment *j*.

*Dimensionless Membrane Permeabilities*

 
*α*_*A*_ = *α*_*A*_′*A*_*M*_/*q*.
 
*α*_AC_ = *α*_AC_′*A*_*M*_/*q*.
 
*α*_*S*_1__ = *α*_*S*_1__′*A*_*M*_/*q*.
 
*α*_*S*_3__ = *α*_*S*_3__′*A*_*M*_/*q*.
 
*α*_*S*_2__ = *α*_*S*_2__′*A*_*M*_/*q*.
 
*α*_H^+^_ = *α*_H^+^_′*A*_*M*_/*q*.
 
*α*_OH^−^_ = *α*_OH^−^_′*A*_*M*_/*q*.

*Other Terms Used in Dimensionless Form*

 
*V*_*R*_ = *V*_(1)_/*V*_(2)_.
 
*γ*_1_ = *K*_*W*_/*K*_*i*__1_.
 
*T* = *qt*/*V*_(1)_.
 
$B_{1}=V_{1}V_{M1}\bar{ChAT}/q$.
 
$B_{2}=V_{2}V_{M2}\bar{AChE}/q$.

## Figures and Tables

**Table 1:** Dimensionless forms of the ordinary differential equations of ChAT inhibition effects by *β*-amyloid aggregates of kinetic mechanism 1.

Item	Compartment	Differential equation
Hydrogen protons	1	$\frac{dh_{\left(1\right)}}{dT}=h_{f}-\gamma_{1}\left(\frac{1}{h_{f}}\right)-\alpha_{\text{H}}\left(h_{\left(1\right)}-h_{\left(2\right)}\right)+\alpha_{\text{OH}}\gamma_{1}\left(\frac{1}{h_{\left(1\right)}}-\frac{1}{h_{\left(2\right)}}\right)$
	2	$\frac{dh_{\left(2\right)}}{dT}=V_{R}\left(\alpha_{\text{H}}\left(h_{\left(1\right)}-h_{\left(2\right)}\right)-\alpha_{\text{OH}}\gamma_{1}\left(\frac{1}{h_{\left(1\right)}}-\frac{1}{h_{\left(2\right)}}\right)-\left(h_{\left(2\right)}-\frac{\gamma_{1}}{h_{\left(2\right)}}\right)+\frac{B_{2}}{k_{h1}}r\left(2\right)\right)$

Acetylcholine (ACh)	1	$\frac{ds_{1(1)}}{dT}=s_{1f}-\alpha_{s1}\left(s_{1(1)}-s_{1(2)}\right)+\frac{B_{1}r\left(1\right)}{K_{s1}}-K_{bA1}s_{1(1)}\text{A}\beta$
	2	$\frac{ds_{1(2)}}{dT}=V_{R}\left(\alpha_{S_{1}}\left({s_{1}}_{\left(1\right)}-{s_{1}}_{\left(2\right)}\right)-{s_{1}}_{\left(2\right)}-\frac{B_{2}r\left(2\right)}{K_{s1}}\right)$

Choline	1	$\frac{d{s_{2}}_{\left(1\right)}}{dT}=s_{2f}+R*s_{2(2)}-\alpha_{S2_{1}}\left({s_{2}}_{\left(1\right)}-{s_{2}}_{\left(2\right)}\right)-\frac{B_{1}}{S_{2\text{ref}}}r\left(1\right)$
	2	$\frac{d{s_{2}}_{\left(2\right)}}{dT}=V_{R}\left(\alpha_{S2}\left({s_{2}}_{\left(1\right)}-{s_{2}}_{\left(2\right)}\right)-\left(1+R\right)*{s_{2}}_{\left(2\right)}+\frac{B_{2}}{S_{\text{2ref}}}r\left(2\right)\right)$

Acetate	1	$\frac{d{s_{3}}_{\left(1\right)}}{dT}={s_{3}}_{f}-\alpha_{S3}\left({s_{3}}_{\left(1\right)}-{s_{3}}_{\left(2\right)}\right)-\frac{B_{1}}{S_{\text{3ref}}}r\left(1\right)$
	2	$\frac{d{s_{3}}_{\left(2\right)}}{dT}=V_{R}\left(\alpha_{S3}\left({s_{3}}_{\left(1\right)}-{s_{3}}_{\left(2\right)}\right)-{s_{3}}_{\left(2\right)}+\frac{B_{2}}{S_{\text{3ref}}}r\left(2\right)\right)$

*β*-amyloid aggregates (A*β*)	1	$\frac{dbA}{dT}=K_{L_{2}}-K_{L_{3}}s_{1(1)}-K_{L_{4}}\text{A}\beta$

Rate of synthesis (*r*(_1_))	1	$r({}_{1})=\frac{\theta_{1}s_{31}s_{21}}{\theta_{2}/h_{1}\left(1+h_{1}\left(1+\text{A}\beta K_{i}\right)+\delta {h_{1}}^{2}\right)+\theta_{3}s_{31}+\theta_{4}s_{21}+\theta_{5}s_{31}s_{21}}$

Rate of hydrolysis (*r*(_2_))	2	$r\left({}_{2}\right)=\frac{s_{12}}{s_{12}+1/h_{2}\left(h_{2}+1+\delta h_{2}^{2}\right)+\alpha s_{12}^{2}}$

**Table 2:** Values of the kinetic parameters.

Parameter	Value	Reference
*θ*_1_	5.2 (0.1)	[5, 6, 29]
*θ*_2_	12	[5, 6]
*θ*_3_	1000	[5, 6]
*θ*_4_	5	[5, 6]
*θ*_5_	1	[5, 6]
α	0.5	[30–34]
δ	1	[30–34]
*k*_*h*1_	1.066 *∗* 10^−6^ kMole/m^3^ (*μ*Mole/mm^3^)	[30–34]
*K*_*s*1_	50.33 *μ*Mole/L	[30–34]
*S*_2ref_	100 *μ*Mole/L	[35]
*S*_3ref_	1 *μ*Mole/L	[35]
*B*_1_	5.033 × 10^−5^ kMole/m^3^ (*μ*Mole/mm^3^)	[30]
*B*_2_	5.033 × 10^−5^ kMole/m^3^ (*μ*Mole/mm^3^)	[30]
α_H^+^_	2.25	[33]
α_OH^−^_	0.5	[33]
α_*S*_1__	1.5	[33]
α_*S*_2__	1.5	[33]
α_*S*_3__	1	[33]
*V*_*R*_	1.2	[33]
pH_*f*_	8.2	[35]
*s*_1*f*_	15	[30]
*s*_2*f*_	1.15	[30]
*s*_3*f*_	3.9	[30]
γ_1_	0.01	[30–34]
*R*	0.8	[36, 37]
*K*_*bA*ref_	20 nM/h	[22, 28]
*K*_*L*2_	Assumed	
*K*_*L*1_	3*∗K*_*L*2_	Assumed
*K*_*L*3_	0.035*K*_*L*2_	Assumed
*K*_*L*4_	0.176*K*_*L*3_	Assumed
*K*_*i*1_	10.015	Assumed
*K*_*bA*1_	0.05	Assumed
*T*_ref_	0.1 h	Assumed
A*β*_ref_	20 nM/L	[23]

## Notations

E_N_: Enzyme N
S_N_: Substrate N (catalyzed by enzyme N)
P_N_: Reaction product N (produced by S_N_ catalyzed by E_N_)
[H^+^]: Hydrogen ions concentration (kmol/m^3^)
[OH^−^]: Hydroxyl ions concentration (kmol/m^3^)
[*S*_1_]: Acetylcholine concentration (kmol/m^3^)
[*S*_2_]: Choline concentration (kmol/m^3^)
[*S*_3_]: Acetyl-CoA concentration (kmol/m^3^)
[*a*]: Acetate concentration (kmol/m^3^)
$\bar{AChE}\text{:}$: Concentration of acetylcholinesterase enzyme in compartment 2 (kg enzyme/m^3^)
$\bar{ChAT}\text{:}$: Concentration of choline acetyltransferase in compartment 1 (kg enzyme/m^3^)
*K*_*s*__1_, *K*_*i*__1_, *K*_*h*__1_: Kinetic constants for the choline acetyltransferase catalyzed reaction (kmol/m^3^)
*K*_*s*__2_, *K*_*i*__2_, *K*_*h*__2_: Kinetic constants for the coenzyme A catalyzed reaction (kmol/m^3^)
*K*_*s*__3_: Kinetic constants for the acetylcholinesterase catalyzed reaction (kmol/m^3^)
*A*_*M*_: Area of membrane separating compartments 1 and 2 (m^2^)
*q*: Volumetric flow rate into compartment 1 (m^3^/s)
*R*_*W*(*j*)_: Rate of water formation in compartment *j* (kmol/m^3^s), *j* = 1 and 2
*r*_(*j*)_: Rate of reaction in compartment *j* (kmol/m^3^s), *j* = 1, 2, and 3
*R*: Recycle flow rate ratio
*V*_(*j*)_: Volume of compartment *j* (m^3^), *j* = 1 and 2
*t*: Time (s)
*α*_H^+^_′: Membrane permeability for hydrogen ions (m/s)
*α*_OH^−^_′: Membrane permeability for hydroxyl ions (m/s)
*α*_*S*_1__′: Membrane permeability for acetylcholine (m/s)
*α*_*S*_2__′: Membrane permeability for choline (m/s)
*α*_*S*_3__′: Membrane permeability for acetyl-CoA (m/s)
*α*_*a*_′: Membrane permeability for acetate (m/s)
*α*_AC_′: Membrane permeability for acetic acid (m/s)
*K*_*W*_: Equilibrium constant for water (kmol^2^/m^6^).

### Abbreviations

AChE: Acetylcholinesterase
ChAT: Choline acetyltransferase
CoA: Coenzyme A
ACh: Acetylcholine
AC: Acetate
CSTR: Continuous stirred tank reactor.

### Subscripts

1: Compartment 1
2: Compartment 2
*f*: Feed condition.
